# Chromatic Adaption of Two Universal Composites: Spectrophotometric Analysis

**DOI:** 10.3390/ma17205103

**Published:** 2024-10-18

**Authors:** Francesca Zotti, Francesca Ferrari, Mattia Penazzo, Giorgia Lanzaretti, Nicoletta Zerman

**Affiliations:** 1 Section of Dentistry and Maxillofacial Surgery, Department of Surgical Sciences, Pediatrics and Gynecology, University of Verona, 37134 Verona, Italy; francesca.zotti@univr.it (F.Z.); nicoletta.zerman@univr.it (N.Z.); 2 Private Practice, 37134 Verona, Italy; ferrari@studiodottoressaferrari.it (F.F.); mattia.penazzo98@gmail.com (M.P.); 3 Private Practice, 36016 Vicenza, Italy

**Keywords:** composite resins, color matching, V class restoration, spectrophotometer

## Abstract

Objective: The purpose of this study was to evaluate the chromatic adaptability and color stability of two different composite resins, Omnichroma and Estelite Bulk-Fill Flow, in Class V restorations at different times. Materials and methods: Standardized Class V cavities were prepared on the labial surface of 34 extracted intact and noncarious human permanent molars. The dental elements were randomly divided into two groups according to the resin composite material. Group 1 was restored with Omnichroma (Tokuyama Dental Corporation Inc., Tokyo, Japan) and Group 2 was restored with Estelite BulkFill Flow Universal (Tokuyama Dental Corporation Inc., Tokyo, Japan). Color properties were assessed using a spectrophotometer, SpectroShadeTM Micro (MHT Optic Research, Niederhasli, Switzerland), at baseline (T0), immediately after Class V restoration (T1), 24 h after restoration (T2) and after thermocycling (T3) (ISO/TS 11405, 2015 protocol). Color difference (ΔE) was analyzed with a Student’s *t*-test and a Wilcoxon–Mann–Whitney test to evaluate the differences, in terms of chromatic adaptability, between the two materials (inter-group analysis) while Kruskal–Wallis test e Two-way ANOVA statistical tests were used to evaluate the color stability of each material over time (intra-group analysis). Results: Regarding the inter-group analysis, there were no statistically significant differences between the two materials in all the comparisons: T0–T1 (*p* = 0.9025), T0–T2 (*p* = 0.2779), T0–T3 (*p* = 0.4694). Moreover, both groups showed an average ΔE > 2. In the intra-group analysis, no statistically significant differences were observed in either Group 1 (*p* = 0.954) or Group 2 (*p* = 0.8654). Conclusions: The in vitro color matching, assessed by spectrophotometry, of the two tested resin composites does not vary at different time intervals. Furthermore, even though both composites use different mechanisms to produce the color the human eye perceives, they show very similar chromatic adaptability.

## 1. Introduction

In recent years, both patients and clinicians have increasingly focused on the esthetic aspect of dental treatments, leading to the development of new materials and techniques to fulfill these cosmetic expectations [1,2,3,4,5,6]. In conservative dentistry, resin-based composite materials have gained widespread recognition [7], and the undeniable progress in the formulation of these materials over time is evident. Manufacturers have actively competed to enhance and address any structural weaknesses that might pose clinical challenges. This includes ensuring a seamless structural and optical integration between the composite restoration, the natural tooth structure, and adjacent teeth [8,9].

Achieving this requires composite resin with varying degrees of opacity and shades to match the subtle color variations in teeth, which can be time-consuming for both the dental practitioner and the patient [10,11]. Furthermore, the clinical efficacy of dental composites depends significantly on their physical, chemical, and mechanical properties, which are strongly influenced by both the oral environment and the inherent characteristics of the resin material [12,13,14].

When light interacts with composite resins, various phenomena can occur, such as light transmission, surface reflection, internal absorption and scattering. Many articles state that the incorporation of colored pigments can significantly affect both the color and translucency of the material that should mimic dental tissue [15]. Additionally, the appearance of the composite restoration is influenced by the layering technique and the thickness of the different composite masses, as well as the cavity depth and the color of the substrate, all of which complicate the accurate selection of the shade needed to achieve a seamless and natural-looking restoration [16].

Recently, universal composite materials have entered the market to simplify the inventory of composite shades, reduce waste, minimize chair-side time, eliminate the need for shade selection, and reduce reliance on shade-matching procedures [10,11,17,18]. Developers claim that the primary advantage of these composites lies in their improved Color Adjustment Potential (CAP), a property defined as the “interaction between the physical and perceptual aspects of blending” [19]. These materials exhibit universal opacity and are available in a limited range of VITA shades. Developers recommend using them in a single shade increment that may effectively match various tooth colors [19]. The resin matrix of these composites primarily consists of Bis-GMA (bisphenol-A glycidyl dimethacrylate) combined in varying proportions with short-chain monomers such as TEGDMA (triethylene glycol dimethacrylate), UDMA (urethane dimethacrylate), Bis-EMA (Bisphenol A polyethylene glycol diether dimethacrylate) and other monomers [20]. The fillers are composed of glass, silica or zirconia with varying filler contents and shapes [11,13,21].

Estelite Bulkfill Flow Universal (Tokuyama Dental Corporation Inc., Tokyo, Japan), as stated by the manufacturer, is a low-viscosity, light-polymerizable, and radiopaque fluid composite. It is equipped with pre-polymerized, spherical supra-nano fillers (average size 200 nm) with a rounded shape, which reduces stress and polymerization shrinkage.

It is primarily indicated for restoring lateral posterior cavities using a layering technique that allows for applying thicknesses of up to 4 mm in a single phase without the need for a final covering layer. After polymerization, Estelite Bulk-Fill Flow changes its translucency from semi-transparent to opaque, increasing its final brightness and enabling a “chameleon-like” effect on dental tissues.

A recent addition to the universal resin-based composites category is Omnichroma from the Tokuyama Dental Corp in Japan. It offers dental practitioners a convenient solution to a common challenge: selecting the appropriate shade. According to the manufacturer, Omnichroma (Tokuyama Dental Corporation Inc., Tokyo, Japan) is a universal shade composite that has advanced chromatic technology governing its optical characteristics. This technology ensures precise reflection of a specific wavelength within the natural tooth-color spectrum [22]. Consequently, it can match all VITA classical A1–D1 shades with a single universal shade. Omnichroma (Tokuyama Dental Corporation Inc., Tokyo, Japan) consists of equal proportions of zirconium dioxide (ZrO_2_) mixed with supra-nanoscale silicon dioxide (SiO_2_) filler particles measuring 260 nm in size and round-shaped composite filler particles with similar characteristics. According to the manufacturer, Omnichroma becomes more translucent after polymerization, with a refractive index of 1.47 before and 1.52 after polymerization. This aligns with previous research identifying a strong correlation between the translucency parameter and the blending effect associated with color adaptation [22]. Once placed in the cavity preparation, this shadeless composite rapidly takes on the color of the underlying and surrounding dentin and enamel, saving both time for the dental practitioner and the patient and eliminating the need for shade selection.

The present in vitro study aims to assess, through spectrophotometry, the chromatic adaptation capability of two different composite resins, Omnichroma (Tokuyama Dental Corporation Inc., Tokyo, Japan) and Estelite Bulk-Fill Flow Universal (Tokuyama Dental Corporation Inc., Tokyo, Japan) in Class V restorations at different timepoints. Specifically, the study aims to:

- Analyze the differences in terms of chromatic adaptation between the two resins at different time intervals (inter-group analysis);
- Analyze the differences in terms of chromatic adaptation for each resin at different time intervals (intra-group analysis).

## 2. Materials and Methods

The sample size was determined using the statistical software G-Power v. 3.1 (University of Düsseldorf; Düsseldorf, Germany). An analysis of statistical significance revealed that a sample size of 33 met the constraints of α = 0.2 and power = 0.95.

To ensure fairness between the two groups, 34 intact upper and lower molar teeth, extracted due to periodontal reasons or lost due to trauma, were collected. They were carefully checked to select teeth that were free of caries, fractures and demineralization. The dental elements were cleaned using curettes, rinsed with 10-volume hydrogen peroxide for 10 s, further rinsed with denatured alcohol for 30 s, and then stored in a saline solution to prevent dehydration.

**Figure 1:**
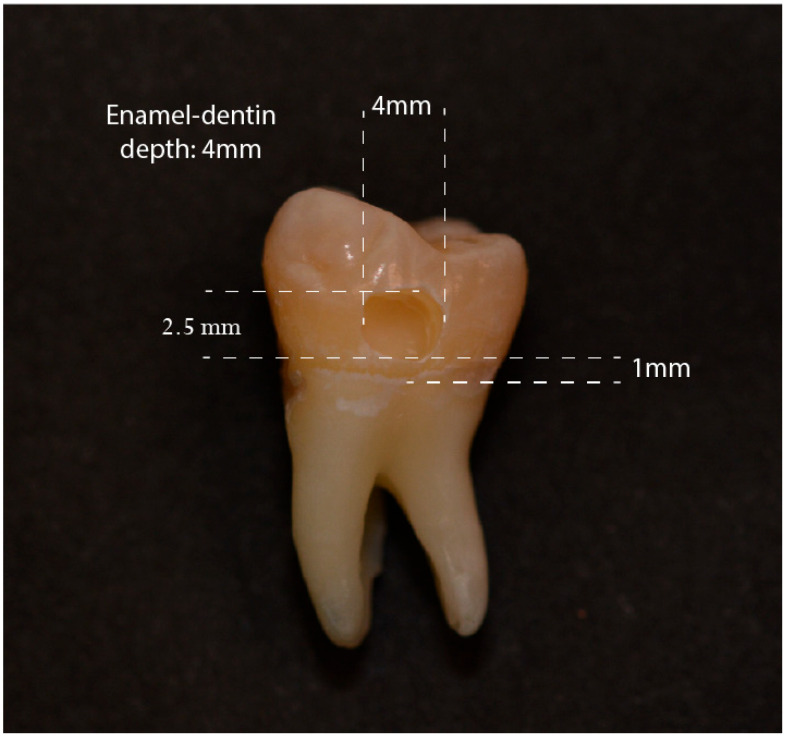
Class V cavity obtained in all dental elements of the sample.

In all 34 molars, a Class V cavity was prepared on the vestibular surface using a cylindrical diamond bur with a thickness of 1 mm under irrigation, as follows (Figure 1):

- Cervical margin located 1 mm from the CEJ (Cementoenamel Junction).
- Enamel–dentin depth of 4 mm.
- Mesio-distal width of 4 mm.
- Corono-apical height of 2.5 mm.

To monitor the cavity depth, a rubber stopper calibrated to the desired depth (4 mm) was placed on the cylindrical bur, and periodic checks of the dimensions were conducted using a periodontal probe. Subsequently, the cavity margins were beveled using a spherical diamond bur with a diameter of 1 mm, utilizing half of its diameter.

The teeth were then randomly divided into two groups of 17 units each using Excel software (Excel, version 18.0, Microsoft Office 2021). The molars of Group 1 were restored using Omnichroma (Tokuyama Dental Corporation Inc., Tokyo, Japan), while those in Group 2 were restored using Estelite Bulk-Fill Flow Universal (Tokuyama Dental Corporation Inc., Tokyo, Japan). The specific procedures were as follows:

- Groups 1 and 2: Selective etching with Tokuyama Etching Gel HV (Tokuyama Dental Corporation Inc., Tokyo, Japan): 30 s on enamel and 15 s on dentin;
- Groups 1 and 2: Rinse for 60 s with water;
- Groups 1 and 2: Adhesive procedure with Tokuyama EE Bond (Tokuyama Dental Corporation Inc., Tokyo, Japan): 10 s on the entire cavity surface and air drying;
- Groups 1 and 2: Polymerization for 20 s according to the manufacturers’ instructions;
- Restoration with:
  ○ Group 1: Incremental layering of Omnichroma composite and polymerization for 20 s for each increment (Group 1);
  ○ Group 2: A single application of Estelite Bulk-Fill Flow Universal followed by polymerization for 20 s (Group 2);
- Groups 1 and 2: Finishing and polishing with 2-step polishing system Enhance PoGo disks (Dentsply Caulk, Milford, DE, USA).

The samples were then thermocycled. Specifically, according to the ISO/TS 11405 (2015) [23] protocol 17th, the sample elements underwent a 30 s process in alternating hot water and cold water for 500 cycles, with temperature variations between 5 ± 2 °C and 55 ± 2 °C, mimicking an aging process of approximately 2 months maximum [20,24].

For the 30 s cycle in water at 55 ± 2 °C, an immersion thermostat was used to maintain a constant water temperature in a 5 L bath, Argo Lab CB 5–10 Immersion Thermostat, Argo Lab, Carpi, Italy). For the 30 s cycle in water at 5 ± 2 °C, a basin containing ice with constant water temperature measurement using a thermometer was used.

The time was measured using a stopwatch; all cycles were completed in 4 h and 10 min. The samples were then stored in saline solution.

To assess chromatic adaption of the two composites, spectrophotometric assessments were performed at the following time intervals:

- T0: Initial color of the tooth after polishing;
- T1: Color immediately after the Class V restoration;
- T2: Color 24 h after the restoration;
- T3: Color after thermocycling procedure.

**Figure 2:**
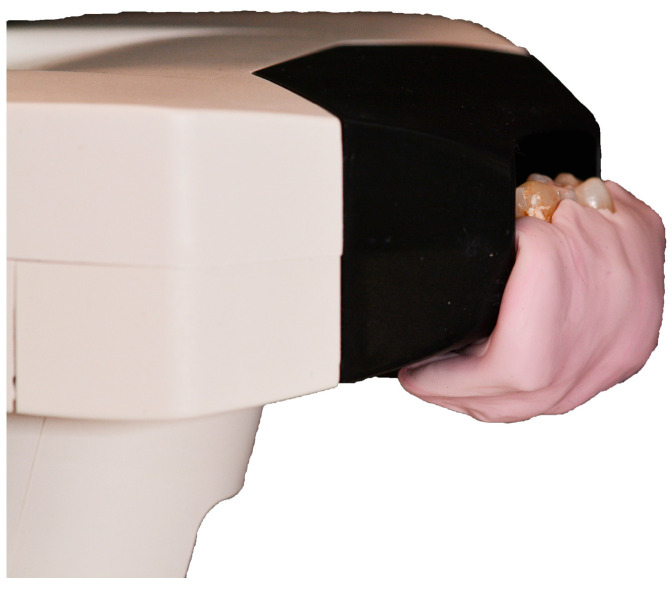
Silicone support used to accommodate the dental elements for the spectrophotometric analysis.

**Figure 3:**
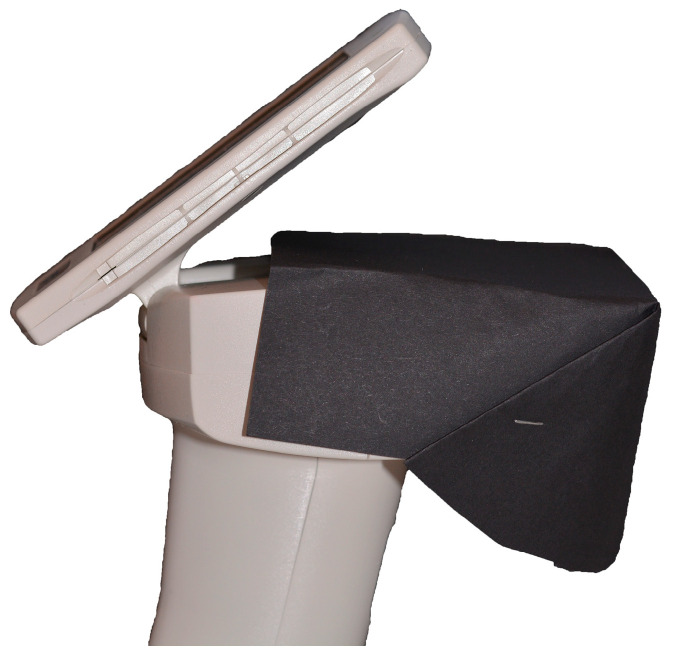
Black cardboard used around the spectrophotometer sensor to simulate the “oral cavity void”.

To perform spectrophotometric evaluation, each tooth was embedded in pink Putty Hard silicone material (Zetalabor, Zhermack Dental, Marl, Germany) to simulate gingival tissue. To standardize and facilitate measurement, the silicone material was molded to allow the dental element examined to be positioned near the spectrophotometer’s sensor (Figure 2). Additionally, the same two adjacent teeth were inserted alongside every element [20,24]. To simulate the “oral cavity void”, a black cardboard was placed around the sample and the sensor (Figure 3).

### 2.1. Inter-Groups Evaluation

**Figure 4:**
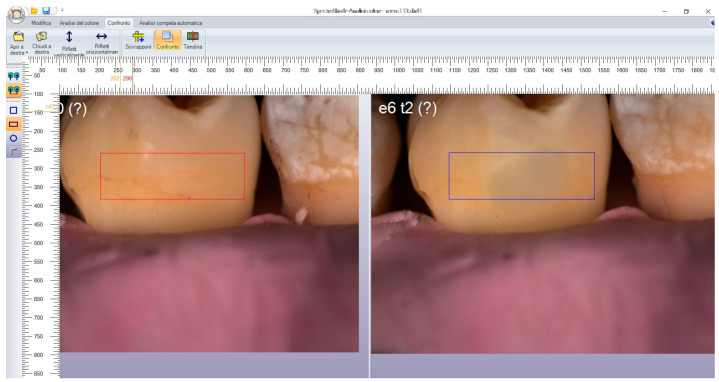
Example of positioning of digital ruler for the selection of a rectangular area comprising sound dental tissue at T0 (red rectangle) and a portion of healthy tissue and a portion of restoration at T2 (blue rectangle).

Since the study’s interest is to highlight the mimetic capability of the two composites, the entire tooth was not selected for image comparison. Instead, a localized area in the middle third/cervical third, comprising a portion of healthy tissue and a portion of the filling, was selected on the software provided with the spectrophotometer (SpectroShadeDatabase^®^, version 3.01). Specifically, using software tools and a digital ruler (ScreenRuler, v.0.10.0, Bluegrams, Peterborough, UK), the same area was selected for each tooth at different timepoints to ensure standardized measurements (Figure 4). Measurements were repeated for each dental element in each group.

The inter-group evaluation was based on comparing the ΔE between time T0 and different time intervals after restoration (T1, T2, and T3) for every element. The following comparisons were carried out:

- ΔE(T0–T1);
- ΔE(T0–T2);
- ΔE(T0–T3).

Potential differences in ΔE values at each interval between the two groups were evaluated as follows:

- ΔE(T0–T1)O vs. ΔE(T0–T1)E;
- ΔE(T0–T2)O vs. ΔE(T0–T2)E;
- ΔE(T0–T3)O vs. ΔE(T0–T3)E.

In the above, “O” denotes the Omnichroma composite and “E” denotes Estelite composite. Before each measurement, spectrophotometer calibration was performed according to the manufacturer’s instructions. Between measurements, the dental elements were consistently stored in saline solution.

### 2.2. Intra-Group Evaluation

The intra-group evaluation, on the other hand, aimed to investigate at what time the two composites exhibited their greatest mimetic capacity. For this purpose, for each tooth in each group, at the time intervals T1, T2, and T3, the ΔE_AB_ was calculated between point A, located within the filling, and point B, located on a portion of healthy tissue, at time intervals T1, T2 and T3 as follows:

- T1ΔE_AB_;
- T2ΔE_AB;_
- T3ΔE_AB_.

Both points were defined with the spectrophotometer software (SpectroShadeDatabase^®^, version 3.01).

**Figure 5:**
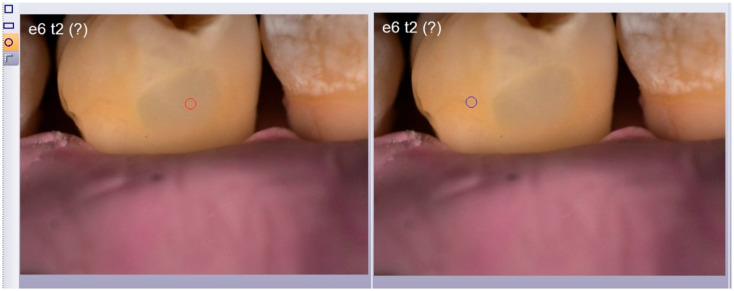
Example of use of the circular selection tool (red and blue circles) provided by the software set to a size of 20 and used to compare point A and point B.

To ensure that the sizes of points A and B remained constant, a circular selection tool provided by the software was used and set to a size of 20 (Figure 5).

Furthermore, to guarantee that the aforementioned points remained in the same position at each time interval, two coordinates (x and y) were identified for each point using a digital ruler (ScreenRuler v.0.10.0, Bluegrams).

For intra-group analysis, differences in the ΔE values at each timepoint were evaluated as follows:

For Group 1:

- T1ΔE_AB_O vs. T2ΔE_AB_O vs. T3ΔE_AB_O

For Group 2:

- T1ΔE_AB_E vs. T2ΔE_AB_E vs. T3ΔE_AB_E

In the above, “O” indicates the Omnichroma composite and “E” indicates the Estelite composite. Before each measurement, a spectrophotometer calibration was performed according to the manufacturer’s instructions. Between measurements, the dental elements were consistently stored in saline solution.

### 2.3. Statistical Analysis

The data ΔE were collected using the software provided with the spectrophotometer, as previously described, and inserted into two Excel tables (Excel, version 18.0, Microsoft Office 2021). (Table 1 and Table 2).

In this study, a ΔE ≤ 2 was chosen as the clinically acceptable value. For the interpretation of the collected data, STATA 16 software (StataCorp, 1985, Los Angeles, CA, USA) was employed. Tests were considered statistically significant for *p* ≤ 0.05.

For the inter-group analysis, the data were tested for normality using the Shapiro–Wilk test, which yielded significance for ΔE values in T0–T2 and T0–T3 but not for the T0–T1 comparison. Therefore, the following tests were employed:

- A Student’s *t*-test to assess the differences in the ΔE(T0–T1) between each group;
- A Wilcoxon–Mann–Whitney test to evaluate the differences in the ΔE(T0–T2) and ΔE(T0–T3) between each group.

For the intra-group analysis, on the other hand, the data were tested for normality using the Shapiro–Wilk test, which showed significance for ΔE values in Group 1 but not for the comparison within Group 2. Therefore, the following tests were used:

- A Kruskal–Wallis test to assess the difference in ΔE for each element within Group 1 at different timepoints;
- A two-way ANOVA to evaluate the difference in ΔE for each element within Group 2 at different timepoints.

## 3. Results

### 3.1. Inter-Group Analysis

The Shapiro–Wilk test yielded the *p*-values included in Table 3.

The Student’s *t*-test was not statistically significant (*p* = 0.9025), indicating that there are no differences in ΔE between the two groups in the first timepoint (T0–T1).

The Wilcoxon–Mann–Whitney tests were not statistically significant (*p* = 0.2779 and *p* = 0.4694), indicating that there are no differences in ΔE between the two groups in the second and third timepoint (T0–T2; T0–T3).

**Figure 6:**
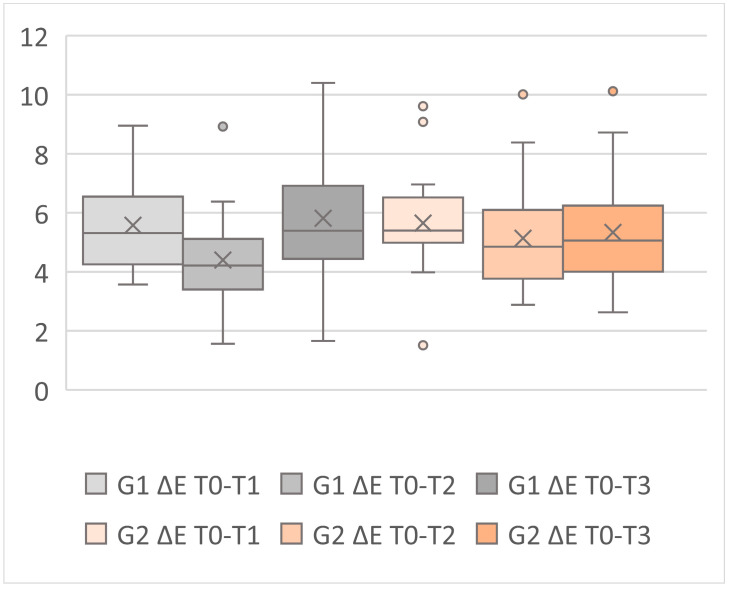
The Box and Whiskers graph is a representation of the inter-group analysis.

Table 1 displays the ΔE values for each dental element in each group at different time intervals, while Table 3 displays the mean and SD values for groups 1 and 2 relative to the ΔE at different time intervals. The data are represented graphically in Figure 6.

### 3.2. Intra-Group Analysis

The Shapiro–Wilk test yielded the *p*-values included in Table 4.

The Kruskal–Wallis test did not provide *p*-values below 0.05 (*p* = 0.954), indicating that there are no differences in the chromatic adaptation (ΔE) of the Omnichroma composite at different time intervals.

The two-way ANOVA test did not yield *p*-values below 0.05 (*p* = 0.8654), indicating no differences in the chromatic adaptation (ΔE) of the Estelite composite at different time intervals.

**Figure 7:**
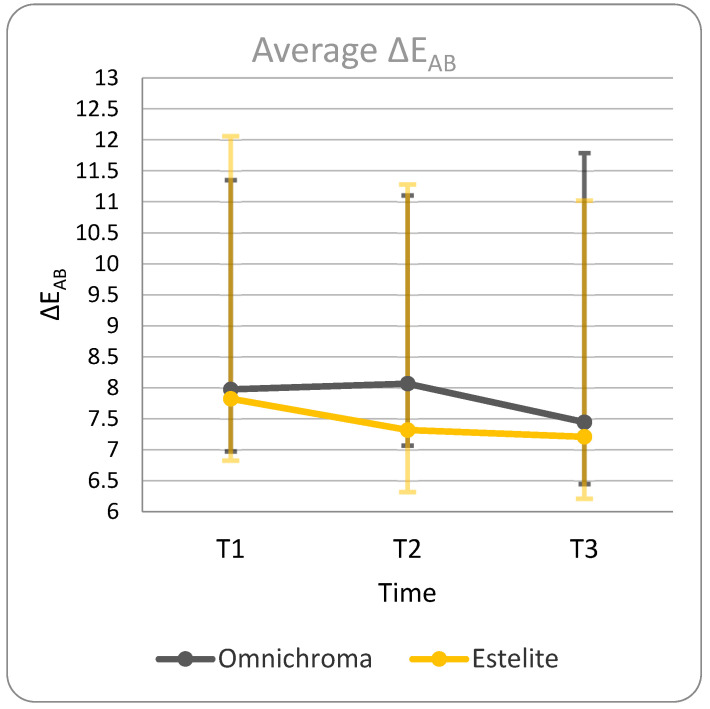
Representation of average ΔE_AB_ values for Group 1 (Omnichroma) and Group 2 (Estelite).

Table 2 displays the ΔE_AB_ between point A, located within the filling, and point B, located on a portion of healthy tooth tissue at different time intervals; Table 4 displays the mean and SD values for groups 1 and 2 relative to ΔE_AB_ at different time intervals. The same data are represented in the graphs in Figure 7.

## 4. Discussion

This in vitro study had the aim of comparing the chromatic adaptability over time of two single-shade dental composites (Omnichroma and Estelite Bulk-Fill Flow, Tokuyama Dental Corporation Inc., Tokyo, Japan). The ambition of the study, in a translational sense, is to assist practitioners in selecting single-shade composites with different characteristics, being aware of their long-term blending capabilities. This opportunity could be a valuable aid in making clinical chair-side decisions that best meet the patient’s esthetic needs, making the most of the properties of the currently available resins. Additionally, the trend in chromatic mimicry over time was evaluated for each composite resin. Omnichroma does not rely on the addition of pigments to generate color; instead, it employs mechanisms to amplify or attenuate various wavelengths by the filler. In contrast, Estelite derives its chromatic properties from the addition of pigments.

This study aims to determine whether these composites can avoid the color-matching phase before performing a restoration, providing a “self-adaptive” composite, or whether reliance on color scales and multi-shade composites remains the gold standard for achieving the highest possible mimicry.

The decision to conduct the study in vitro was driven by the desire to analyze standardized posterior tooth restorations in terms of shape and size to provide consistent and repeatable results regarding the physical properties of the resins used, which would be impossible to achieve in vivo.

Given that most studies in the literature employ customized thermocycling procedures, demonstrating a definite consistency in temperature selection (5–55 °C) but significant variability in terms of the number of cycles and the duration of each cycle [1], in this study, a standardized procedure was chosen. The procedure used for this research, ISO TR 11405 standard (2015), consisted of 500 cycles of 30 s in water with temperature variations between 5 ± 2 and 55 ± 2 °C as an appropriate artificial aging method for samples, simulating a 2 month aging process [2].

Furthermore, since the study’s intention was not to investigate the presence of micro-gaps at the interface or the mechanical properties of the composite resins, it was deemed sufficient, based on the existing literature, to simulate short-term aging (approximately two months).

The SpectoShade^TM^ Micro (MHT Optic Research, Niederhasli, Switzerland) has demonstrated high reliability and accuracy compared to other spectrophotometers on the market [3]. Furthermore, it exhibits good measurement repeatability and an 82.7% congruence with the Vita Classical scale [4].

A systematic literature review conducted by Chen et al. (2012) [5] revealed that instrumental color measurements performed with a spectrophotometer can ensure exact and accurate color matching results. Although spectrophotometers are primarily used for color detection in anterior fields, in vitro their use has also been possible for posterior fields.

Currently, there is no unanimous consensus regarding the ΔE threshold beyond which the human eye can perceive a color difference between two materials. Many studies consider a ΔE value of 3.0 [6] and highly cited research in the literature reports a ΔE value of 2.7 [7].

According to other studies, only ΔE values < 1 are entirely imperceptible to the human eye, whereas if 1 < ΔE < 2, only highly trained observers can perceive differences, but the result remains clinically acceptable. A ΔE > 2 would be perceived even by those without trained eyes and is therefore clinically unacceptable [8,9]. Thus, in this study, a ΔE value of 2 was chosen as the threshold. In the context of medical analysis, the provided text can be translated as follows: Regarding inter-group analysis, no statistically significant differences were observed between the two materials in either the T0–T1 comparison (*p* = 0.9025), the T0–T2 comparison (*p* = 0.2779), or the T0–T3 comparison (*p* = 0.4694). This suggests that both composites, despite utilizing two different mechanisms for generating the color perceived by the human eye, behave very similarly.

Analyzing the average ΔE value for each group at the three timepoints (Table 3); Figure 6) yielded the following results:

- In Group 1, an average ΔE value > 2 was observed in every comparison, which, according to the study’s threshold, indicates a clinically unacceptable result. The best result was obtained in the second comparison after storing the sample for 24 h in a physiological solution. It is worth noting an increase in ΔE values at the T0–T1 and T0–T3 intervals, suggesting that the color stability of the Omnichroma composite appears to decrease after thermocycling but benefits from a 24 h restoration period. This is likely due to water absorption by the resin, which improves its color properties.
- In Group 2, an average ΔE value > 2 was observed in every comparison, which indicates a clinically unacceptable result according to the study’s threshold. The best result was obtained in the second comparison after storing the sample for 24 h in saline solution, likely for the same reasons mentioned above. However, the average ΔE values for Estelite restorations consistently decreased over different intervals.

Regarding intra-group analysis, the initial hypothesis expected a progressive decrease in ΔE_AB_ between T1 and T2 and a further decrease in stability of ΔE_AB_ between T2 and T3. In this case, the predicted result involved ΔE_AB_ values close to 2 (≈2) for both groups.

Statistical analysis did not reveal statistically significant differences in measurements at different time intervals within either Group 1 (*p* = 0.954) or Group 2 (*p* = 0.8654).

Both composites, therefore, do not significantly change their color stability in the short term. A similar result is reported in the study by Zulekha et al. (2022) [10].

Analyzing the average ΔE_AB_ value for each group at the three timepoints (Table 4; Figure 7) yielded the following results:

- In Group 1, there was a progressive decrease in the average ΔE_AB_ value, with lower values recorded after the thermocycling procedure. However, an average ΔE_AB_ > 2 was recorded in every comparison, indicating a clinically unacceptable result according to the study’s threshold.
- In Group 2, there was a progressive decrease in the average ΔE_AB_ value, with lower values recorded after the thermocycling procedure. However, an average ΔE_AB_ > 2 was recorded in every comparison, indicating a clinically unacceptable result according to the study’s threshold.

Furthermore, a high standard deviation was observed in both groups, indicating significant heterogeneity in the ΔE_AB_ values within the sample. This could be attributed to the initial color of the dental element, as it has been demonstrated that single-shade composites adapt better to shades with higher values compared to those with lower values [12]. The fact that the sample consists of molar elements, which typically have lower values and higher saturation than anterior teeth, may be related to this conclusion.

In both groups and both analyses, there is a noticeable decrease in the recorded ΔE from T1 to T2, which then remains relatively constant at T3. This can be explained by the fact that a 30 min dehydration of the dental element, necessary for the restorative procedure, is considered sufficient to create a clinically significant color change. Therefore, once the tooth has rehydrated, there will be a decrease in ΔE and thus improved mimicry of the composite. Additionally, it should be noted that every composite resin benefits from a helpful time interval after restoration for water absorption, which enhances its color properties [19,21].

The limited literature available about single-shade composites presents conflicting results regarding the mimetic capacity of Omnichroma. A study by de Abreu et al. (2021) [11] reports better chromatic adaptation of multi-shade composites compared to single-shade composites (evaluating Class III through photographic and visual analysis). On the other hand, according to the study by Pereira Sanchez N. et al. (2019) [13], carried out on Class I restorations, Omnichroma had the lowest chromatic mismatch. This may appear to contradict the results of the current study; however, it should be noted that the analysis in de Abreu et al.’s study was based on visual evaluation rather than spectrophotometric assessment.

The study by AlHabdan A. et al. (2022) [22], performed with spectrophotometric analysis on anterior teeth, reports an average ΔE of 6.474 between the virgin tooth and the same tooth after restoration, which is a relatively high ΔE. This aligns with the results of the current study, particularly regarding the second analysis performed.

There is limited literature available regarding the chromatic adaptation of Estelite Bulk-Fill flow Universal. However, its chromatic behavior can be attributed, in terms of single-shade bulk-fill composites on the market, to other pigmented products of the same category.

In the present study, restorations using both composites never resulted in an average ΔE ≤ 2 when comparing the tooth at time T0 to the restored tooth. From a clinical perspective, this suggests that even less experienced human observers would likely be able to discern the area where the filling is located. Therefore, the esthetic outcome is not clinically acceptable. This is a crucial consideration because the threshold values for ΔE that indicate mimicry to the human eye have been highly variable in the up-to-date literature. Consequently, conducting a parallel study that combines ΔE values with subjective evaluation on a sufficiently large sample would be advisable for establishing a reference ΔE value that can be used for future comparisons.

As reported in the study by Bompolaki D. [25], single-shade resin composites appear to simplify the color selection process but are more suitable for small restorations and monochromatic elements. In this study, Class V cavities of posterior teeth were used, which are by definition volumetrically larger: this factor could have certainly influenced the ability to blend in. The translucency of single-shade composites closely resembles that of enamel, and they appear to be more effective at matching lighter shades compared to darker ones. These composites also tend to achieve a smooth finish with ease and retain their gloss over time. In small to medium-sized restorations, they exhibit excellent blending properties and become nearly undetectable, particularly when encased entirely by the surrounding tooth structure [25].

One limitation of the present study could be the cavity size, which extends for 4 mm mesiodistally and 4 mm in depth; this could limit the mimicry capability of the composites, especially considering that posterior teeth cavities were selected.

Another limitation can be observed in retrieving areas and points A and B for the two analyses (especially in the intra-group evaluation). Despite using a digital ruler (ScreenRuler v.0.10.0, Bluegrams) and customized silicone masks, and even with the same operator, in some cases, perfect alignment between images at different timepoints could not be achieved. This is likely due to minor errors in the positioning of the dental element during measurement and manual selection of the area and operator’s position.

## 5. Conclusions

The results of this study indicate that the in vitro chromatic adaptation of the two tested resins, measured using spectrophotometry, does not vary across different time intervals. Additionally, despite utilizing two different mechanisms for generating the color perceived by the human eye, both composites behave very similarly in terms of chromatic mimicry.

Single-shade composites may find more predictable applications in smaller-sized restorations. It is crucial to establish a correlation between ΔE obtained through spectrophotometry and the perception of mimicry by the human eye, at least on average. This will allow for translational results in chromatic adaptation analyses.

## Figures and Tables

**Table 1:** Names and ΔE values at each interval for every element of the two groups.

		ΔE T0–T1	ΔE T0–T2	ΔE T0–T3
Group 1 (Omnichroma)	O1	5.97	4.21	6.75
	O2	4.22	2.59	5.39
	O3	6.52	5.26	10.4
	O4	8.95	8.92	9.66
	O5	6.37	4.9	5.82
	O6	3.57	3.22	4.65
	O7	4.58	3.58	4.16
	O8	7.68	6.38	9.96
	O9	5.31	3.92	6.52
	O10	6.65	4.97	5.7
	O11	5.2	4.52	4.4
	O12	6.58	5.69	7.08
	O13	4.22	1.56	2.39
	O14	3.99	3.74	4.48
	O15	6.11	4.4	4.65
	O16	4.29	4.09	5.2
	O17	4.64	2.82	1.66
Group 2 (Estelite)	E1	3.98	2.88	3.31
	E2	5.4	3.65	3.85
	E3	6.23	5.29	5.15
	E4	6.96	3.06	2.63
	E5	4.94	5.4	4.58
	E6	5.1	4.17	5.62
	E7	5.08	4.15	4.58
	E8	6.81	6.28	5.83
	E9	4.11	5.58	4.16
	E10	9.61	10.01	10.12
	E11	5.61	6.16	6.74
	E12	5.51	6.03	6.66
	E13	5.03	4.47	4.9
	E14	5.26	3.88	5.06
	E15	9.08	8.38	8.72
	E16	5.83	4.85	5.25
	E17	1.51	3.06	3.56

**Table 2:** ΔE_AB_ values for each element of both groups at three time intervals.

		T1 ΔE_AB_	T2 ΔE_AB_	T2 ΔE_AB_
Group 1 (Omnichroma)	O1	3.98	2.88	5.12
	O2	5.4	3.65	12.14
	O3	6.23	5.29	18.96
	O4	6.96	3.06	7.37
	O5	4.94	5.4	7.3
	O6	5.1	4.17	7.5
	O7	5.08	4.15	14.23
	O8	6.81	6.28	5.02
	O9	4.11	5.58	10.6
	O10	9.61	10.01	6.56
	O11	5.61	6.16	2.11
	O12	5.51	6.03	3.95
	O13	5.03	4.47	7.35
	O14	5.26	3.88	5.32
	O15	9.08	8.38	2.51
	O16	5.83	4.85	5.18
	O17	4.96	6.44	5.33
Group 2 (Estelite)	E1	4.34	2.53	4.55
	E2	4.11	8.13	4.79
	E3	5.12	4.5	4.01
	E4	10.09	6.67	7.31
	E5	8.74	6.36	6.18
	E6	13.22	11.47	11.86
	E7	13.88	14.81	13.17
	E8	11.38	12.03	9.52
	E9	12.29	10.7	10.57
	E10	4.76	6.16	4.89
	E11	6.39	5.39	5.03
	E12	3.07	3.67	2.53
	E13	5.84	6.28	3.91
	E14	12.51	10.81	12.07
	E15	12.85	11.41	13.74
	E16	2.88	2.22	6.1
	E17	1.52	1.22	2.32

**Table 3:** Mean values, SD values for each group at different time intervals and *p*-values for every comparison.

	Mean Value	SD	*p*-Value (O vs. E)
ΔE T0–T1 O	5.579412	1.455268	0.16154
ΔE T0–T1 E	5.65	1.852849	
ΔE T0–T2 O	4.419412	1.636782	0.01467
ΔE T0–T2 E	5.135294	1.898223	
ΔE T0–T3 O	5.815882	2.433663	0.02580
ΔE T0–T3 E	5.336471	1.90412	

**Table 4:** Mean, SD and *p*-values for groups 1 and 2 related to ΔE_AB_ at different time intervals.

		Mean Value	SD	*p*-Value
Group 1 (Omnichroma)	T1ΔE_AB_O	7.971765	3.378611	0.03289
	T2ΔE_AB_O	8.065882	3.037476	0.37219
	T3ΔE_AB_O	7.444118	4.343725	0.02314
Group 2 (Estelite)	T1ΔE_AB_E	7.822941	4.235149	0.07312
	T2ΔE_AB_E	7.315294	3.964138	0.50232
	T3ΔE_AB_E	7.208824	3.810713	0.06379

## Data Availability

The original contributions presented in the study are included in the article, further inquiries can be directed to the corresponding author.
